# Effects of orthographic transparency on rhyme judgement

**DOI:** 10.3389/fpsyg.2023.1038630

**Published:** 2023-03-06

**Authors:** Jia’en Yee, Ngee Thai Yap, Rozi Mahmud, M. Iqbal Saripan

**Affiliations:** ^1^Faculty of Modern Languages and Communication, Universiti Putra Malaysia, Selangor, Malaysia; ^2^Faculty of Medicine and Health Sciences, Universiti Putra Malaysia, Selangor, Malaysia; ^3^Faculty of Engineering, Universiti Putra Malaysia, Selangor, Malaysia

**Keywords:** rhyme judgment, phonological awareness, orthographic transparency, cross-language transfer, multiliteracy, multilingualism

## Abstract

This study investigated the influence of multiliteracy in opaque orthographies on phonological awareness. Using a visual rhyme judgement task in English, we assessed phonological processing in three multilingual and multiliterate populations who were distinguished by the transparency of the orthographies they can read in (*N* = 135; ages 18–40). The first group consisted of 45 multilinguals literate in English and a transparent Latin orthography like Malay; the second group consisted of 45 multilinguals literate in English and transparent orthographies like Malay and Arabic; and the third group consisted of 45 multilinguals literate in English, transparent orthographies, and Mandarin Chinese, an opaque orthography. Results showed that all groups had poorer performance in the two opaque conditions: rhyming pairs with different orthographic endings and non-rhyming pairs with similar orthographic endings, with the latter posing the greatest difficulty. Subjects whose languages consisted of half or more opaque orthographies performed significantly better than subjects who knew more transparent orthographies than opaque orthographies. The findings are consistent with past studies that used the visual rhyme judgement paradigm and suggest that literacy experience acquired over time relating to orthographic transparency may influence performance on phonological awareness tasks.

## 1. Introduction

An important question in the research on reading is how the orthographic properties of languages affect the processes that underlie visual word recognition, which involves making associations between orthography and phonology (e.g., Patterson et al., 1996). However, orthographies vary in the level of consistency between graphemes and phonemes. Orthographies with a high degree of consistency are considered transparent and more easily decoded than opaque orthographies, which have more unpredictable correspondences. These differences in grapheme-to-phoneme correspondences (GPCs) across orthographies affect the contribution of phonological processing skills. Evidence has shown that opaque orthographies require more phonological processing resources and greater phonological awareness (PA) than transparent orthographies (Vigneau et al., 2006). For instance, opaque orthographies consist of rhymes with incongruent orthographic endings (e.g., *night*/*kite*) that would not be possible in transparent orthographies. Neuroimaging studies have corroborated this additional layer of processing difficulty by showing that processing an orthography with more inconsistencies between its symbols and sounds triggers substantially more activation across a wider range of brain regions. This is indicative of greater demands on orthographic and phonological processing for opaque orthographies than transparent orthographies (Peng et al., 2004; Bolger et al., 2008). The experience of processing opaque orthographies enables bilinguals to become more sensitive to the relationship between representations in orthography and phonology. Furthermore, by assessing PA using measures such as the rhyme judgement task, Rudner et al. (2019) showed that the orthographic representations of one’s language can influence phonological processing. Specifically, cross-language transfer of PA between orthographies of different transparencies has been observed. However, the findings in the field are seemingly mixed, as the transfer of PA has been observed in both directions between transparent and opaque orthographies (Durgunoğlu et al., 1993; Chiang and Rvachew, 2007). While some studies have observed PA transfers from transparent to opaque orthographies, others have found the reverse. Additionally, these studies have largely been conducted with monolinguals and bilinguals, and it is unclear how PA is influenced in multilinguals. To our best knowledge, no study has investigated the influence of opaque literacy experience on PA within a population that is multiliterate in multiple orthographies of varying transparency. To do so, this study tested three groups of multilinguals with varying opaque literacy experience using a visual rhyme judgement task in English consisting of both congruent and incongruent conditions.

## 2. Literature review

### 2.1. Orthographic transparency

Orthographic structures across languages map onto phonological systems, with remarkable differences in the level of regularity and consistency between their written representations and speech sounds (Protopapas and Vlahou, 2009). This degree of correspondence is referred to as orthographic transparency or sometimes as orthographic depth (Katz and Frost, 1992). Decoding orthographies of varying levels of regularity between graphemes and phonemes demands different sets of cognitive and linguistic skills, with opaque orthographies requiring more resources than transparent orthographies (Seymour et al., 2003; Bolger et al., 2008; Borleffs et al., 2017, 2019). Transparent orthographies tend to have a one-to-one correspondence between spelling and sound, where any given letter or grapheme is pronounced the same regardless of where it appears in a word or the word it appears in (Winskel and Lee, 2013). Thus, dissecting words into their phonemic components is relatively easy in transparent orthographies (Liberman, 1973). Examples of transparent orthographies include Arabic, Italian, Greek, and Malay. On the other hand, opaque orthographies slide toward the opposite end of the univalent to multivalent spectrum, exhibiting multivalent spelling-to-sound mappings where GPCs are more irregular and inconsistent. In opaque orthographies, a symbol may be pronounced in multiple ways, and a sound may be orthographically represented in multiple ways. Therefore, sounds in opaque writing systems are not as reliably predicted based on the orthographic structure as they are in transparent orthographies. Examples of opaque orthographies include Chinese, Danish, English, and French (Share, 2008). Owing to ambiguous mapping between sound and spelling in opaque orthographies, many words cannot be pronounced correctly without learning their pronunciation. Chinese orthography is considered highly opaque, where the pronunciation of its units is mostly unpredictable (Weekes et al., 2008). The sounds of Chinese characters are sometimes predictable from their phonetic radicals, but the same radical would be pronounced completely differently when found in other characters. Hence, to pronounce certain characters, one would need to memorize the entire character and retrieve its sound by accessing one’s lexicon (Frost, 2005). The orthographic transparency of a language thus affects the way words are processed, and is suggested to be key in modulating the necessary phonological processing skills (Aro and Wimmer, 2003). More specifically, opaque orthographies require more phonological access and manipulation than transparent languages (see Van Orden, 1987; Bitan et al., 2009; Caravolas et al., 2013).

### 2.2. Orthographic transparency and phonological awareness

Underlying the process of mapping orthographic symbols to sounds (and vice versa) is PA (Share, 1995; Torgesen et al., 1999; Foy and Mann, 2006). This is the ability to analyze, manipulate, and segment smaller sound units in spoken words and can be influenced by literacy training (de Gelder and Vroomen, 1992; Smith et al., 2014). Moreover, PA is known to be a significant predictor of reading abilities across a range of languages, such as Arabic (Tibi and Kirby, 2018), Italian (Cossu et al., 1988), Greek (Loizou and Stuart, 2003), and Swedish (Kjeldsen et al., 2014). For instance, in a longitudinal study spanning 9 years, Kjeldsen et al. (2014) observed that PA training was associated with enhanced decoding skills and reading comprehension in a group of 209 Swedish-speaking children. Additionally, PA was found to not only predict the reading of transparent orthographies but also opaque orthographies such as French, English (Plaza and Cohen, 2007), and even Chinese, which is highly opaque and non-alphabetic (Newman et al., 2011). Notably, PA is more heavily depended upon when reading in opaque orthographies. For instance, a study conducted with children found that in learning to read transparent orthographies, PA was a significant predictor in the early years, whereas its significance persisted beyond the early years for opaque orthographies (Furnes and Samuelsson, 2010). Additionally, greater PA better predicts early development of more opaque languages like English, but not of languages with transparent orthographies (Georgiou et al., 2008). Its importance in opaque orthographies is evident even in atypical populations, where PA impairments compromise the decoding of transparent orthographies less than opaque orthographies (Landerl et al., 2013, 2019). This was suggested to be due to the greater demands placed on PA in opaque orthographies, where each symbol has more than one pronunciation (Furnes and Samuelsson, 2010).

### 2.3. Cross-language transfer of phonological awareness

Up to this point, this paper has reviewed the evidence for PA’s influence on decoding. However, it is important to also note that the influence between the two is bidirectional (Perfetti et al., 1987; Alcock et al., 2018; Landerl et al., 2019), and that PA is a metalinguistic skill also influenced by literacy experience. This has been noted in studies of monolingual and bilingual populations in various languages (Ehri, 1989; Morais, 1991; Castles et al., 2003; San Francisco et al., 2006). Furthermore, past studies with low-literacy adults in different languages have shown that exposure to speech alone without the written form may not adequately improve PA (Jiménez and Venegas, 2004; Bar-Kochva et al., 2021). For example, without access to the written form of their native language, adult Somali learners of English struggled with correspondences between symbols and sounds (Kurvers et al., 2010). These studies show a close connection between orthographic information and phonological processing, where orthographic knowledge influences PA.

In addition, cross-linguistic studies have proposed that orthographic knowledge can facilitate PA transference across languages. Some have posited that PA transference depends on the degree of congruency in the phonological structures of both languages (Yeong and Rickard Liow, 2012), and that it only facilitates in transfers from transparent orthographies like Spanish to less transparent orthographies like English (Durgunoğlu et al., 1993). However, considerable research has also shown otherwise for language pairs such as English and French (Chiang and Rvachew, 2007), English and Arabic (Wagner et al., 1989), and English and Italian (Cossu et al., 1988), among others, where PA in the more opaque orthography explains literacy in the more transparent orthography. For example, Chiang and Rvachew (2007) found that in a group of bilingual children, PA in French (relatively less opaque) was predominantly explained by PA in English (relatively more opaque). Interestingly, the cross-language transfer of PA has also been documented in the direction of non-alphabetic, opaque orthographies to alphabetic, transparent orthographies. For example, this was observed to occur from Chinese (a logographic, opaque orthography) to English and Dutch (Gottardo et al., 2001; Yuan et al., 2020) as well as bidirectionally between Cantonese and English (Chan, 2018). Chan’s study found that training in Cantonese PA improved English PA and vice versa on measures of rhyme oddity and phoneme deletion. In Gottardo et al.’s (2001) study, competence in detecting Chinese rhyme was predictive of word reading, word identification, and rhyme detection in English, a language relatively less opaque than Chinese. Such observations were expounded alongside theories of cross-language transfer and structural sensitivity.

At its simplest, cross-language transfer theory predicts that learning one language supports the learning of another if their shared linguistic features are more apparent or complex in the former (Osgood, 1949; Kuo et al., 2016). In terms of orthographic transparency, opaque literacy experience could have a facilitative effect on a less opaque orthography, where PA transference can occur from the language with a more complex PA component to one that is simpler (i.e., highly regular GPCs). In other words, literacy in opaque orthographies involves managing more complex phonological segments, which could increase sensitivity to more varied and inconsistent GPCs across languages. Furthermore, structural sensitivity theory by Kuo and Anderson (2012) posits that consistent exposure to more than one language may enable individuals to more readily restructure language input and assign linguistic structures because they encounter more varied linguistic segments. Literacy experience in more opaque orthographies may therefore facilitate the ability to separate phonological units from their original contexts more flexibly and apply this skill to other languages. In conjunction with past studies conducted with monolingual and bilingual populations, these two theories highlight how more opaque literacy experience supports PA in less opaque orthographies. However, the influence of orthographic knowledge has not been investigated extensively in multilinguals, where individuals could be literate in orthographies with different levels of transparency. It is thus unclear how literacy in more opaque orthographies relying significantly more on PA would influence PA.

### 2.4. Rhyme judgement

The rhyme judgement task has long been used to examine phonological processing across various orthographies due to its greater demand on PA (Stuart-Smith and Martin, 1999; Brennan et al., 2013). Awareness of rhymes is a fundamental component of PA, a foundational step for progressing toward sharper awareness of syllables and phonemes (Jing et al., 2019). Rhyme judgements require access to phonological representations and are affected by orthographic systems (Bolger et al., 2008). In visual word rhyming tasks, subjects are typically shown word pairs that may or may not be orthographically similar and asked to decide if they rhyme. Phonological processing speed is associated with the quality of the phonological representations, such that faster processing indicates better-preserved phonological representations (Andersson, 2002). Opaque orthographies such as English include rhyming words consisting of both congruent and incongruent rhymes; some share similar orthographic endings (*peach*/*beach*), while others are spelled differently but are phonologically similar (*weep*/*leap*). In contrast, no such inconsistencies exist in transparent orthographies (e.g., in Malay: *sudu* and *madu*). Regular GPC in transparent orthographies facilitates rhyme judgements as there is no phonological or orthographic interference. In other words, when the orthography of words is consistent with their phonology, individuals make better rhyme judgements (Sterne and Goswami, 2000). To our best knowledge, investigations on visual rhyme judgements have not been conducted within a population that is multiliterate in orthographies of varying transparency. This study aimed to determine whether more opaque literacy experience (i.e., literacy in more than one opaque orthography) facilitates PA.

### 2.5. Orthographies relevant to the current study

A variety of mother tongues or home languages are used in Malaysia, with English and Malay formally taught in preschools and primary schools. Additionally, many individuals are literate in and/or speakers of multiple languages, such as Arabic, Cantonese, Hindi, Mandarin, and Tamil. Therefore, different profiles of multilinguals with different levels of exposure to opaque orthographies are commonly found. The following paragraphs further describe the orthographic properties of English and Malay, in which all subjects in this study are literate, as well as Arabic and Chinese, in which some subjects are also literate.

#### 2.5.1. Transparent orthographies involved

Arabic belongs to the Afro-Asiatic language family and is considered to have a transparent orthography. Like Persian and Hebrew, Arabic uses a consonantal system written in the abjad script and has two forms: one includes diacritics and the other does not. The Arabic alphabet contains 28 characters, 6 vowels, 30 consonants, and 36 phonemes (Perfetti and Harris, 2013). Diacritics are added to indicate vowels in the early stages of language acquisition (Ravid and Haimowitz, 2006), aiding in sounding out words. On the other hand, the un-vowelized version tends to create ambiguity as various words can be formed from the same set of consonants depending on which vowels are filled in. This makes Arabic orthography transparent in the phoneme-to-grapheme direction but less so in the grapheme-to-phoneme direction.

Malay belongs to the Austronesian language family and is the national language of Malaysia. It is the home language, or L1, for most Malaysians and is formally taught in school. Like Arabic, its orthography is transparent with regular, consistent GPCs. Graphemes and phonemes have a one-to-one correspondence, making them easier to learn than English (Rickard Liow and Lee, 2004). It has two writing systems or scripts: the Latin alphabet (Rumi) and Arabic alphabet (Jawi). The Latin script is used widely in Malaysia and has 26 letters, as in English.

#### 2.5.2. Opaque orthographies involved

The two opaque orthographies in this study are English and Chinese, the former being less opaque than the latter. English orthography consists of 26 letters, 5 vowels, and 21 consonants forming 40 phonemes, and is considered opaque owing to irregular GPCs. English vowel pronunciation is estimated to only be consistent across words 51% of the time (Treiman et al., 1995). Six of the 12 digraphs in English have varied pronunciations, depending on their position in a word (Ellis et al., 2004). Furthermore, several letters are pronounced differently depending on where they appear in a word and the word they appear in. For instance, “a” in *apple* is different from “a” in *watch*. Additionally, a single sound may be written in different ways. For example, the word *leap* is phonologically consistent with *keep* despite different orthographic endings.

Compared to English, Chinese orthography is even more opaque. It uses a morphosyllabic script with square characters consisting of strokes and radicals (semantic and phonetic) that are not perfectly reliable in indicating pronunciation (Hsiao and Shillcock, 2006). Radicals in characters hint at the character’s pronunciation in some cases but not others. For example, some Chinese characters share the same radical but their pronunciation differs vastly. The character “鞋” is pronounced /xie2/while “蛙,” which shares the same radical, is pronounced /wa1/ (the number indicates tone). Irregularities in the phoneme-to-grapheme direction are even more prevalent: on average, five different characters represent one tone syllable (Chen and Pasquarella, 2017). Furthermore, there are characters with more than one pronunciation called polyphonic characters (Yang et al., 2021). Examples of polyphonic characters include “了,” which can be pronounced /le4/or/liao/, and “差,” which can be pronounced in four ways: /cha1/, /cha4/, /chai1/, and/ci1/. Even when radicals are useful in sounding out characters, they may not reveal the character’s full pronunciation. In other words, phonology is mostly mapped to orthography at the whole-character level (Deng et al., 2013).

## 3. This study

As it has been proposed that bilinguals enjoy a cognitive advantage over monolinguals due to their frequent engagement of executive control, a cognitive function also involved in language control (Costa et al., 2006; Bialystok, 2011), it would be plausible to hypothesize that greater exposure to and skill in speech sounds from opaque orthographies would form a consistent practice that enhances PA. This investigation aimed to shed light on how PA could be improved in multilingual adults, especially given PA’s importance in speech, listening, and reading. We expected that subjects experienced with an additional opaque orthography (e.g., Chinese) apart from English would perform better than 1) subjects inexperienced with an opaque orthography other than English and 2) subjects experienced with an additional transparent orthography (i.e., Arabic). We expected a main effect of phonological similarity, where performance in rhyming conditions would be better than in non-rhyming conditions. It was also hypothesized that a main effect of orthographic similarity would be found, in that orthographic congruence facilitated performance. This is attributed to the lower cognitive demand required to process rhymes compared to non-rhymes (Coch et al., 2008) and the facilitative effect that congruent orthography has been found to have in past research (Damian and Bowers, 2010). Additionally, we expected to find an interaction effect between phonological and orthographic similarities. Specifically, accuracy and response time (RT) for rhyming word pairs with different orthographic endings (O−P+) and non-rhyming word pairs with similar orthographic endings (O+P−) were expected to be poorer than for rhyming word pairs with matching orthographic endings (O+P+) and non-rhyming word pairs with different orthographic endings (O−P−). This expectation is based on past research, which has shown that it is more challenging to manage incongruent conditions than congruent ones (Andersson, 2002).

## 4. Materials and methods

This study obtained informed consent from all subjects and was approved by the Ethics Committee for Research Involving Human Subjects at Universiti Putra Malaysia (JKEUPM2020-272).

### 4.1. Subjects

Three groups of 45 participants each, who reported using at least two languages (*M* = 2.97, *SD* = 0.72), were recruited to perform the tasks online. All were literate in English and Malay, and some were literate in additional languages. There were two transparent groups. The first consisted of subjects literate in languages written in the Latin script that were all transparent apart from English. The second consisted of subjects literate in languages written in both Latin and Arabic scripts that were all transparent, apart from English. The opaque group consisted of subjects literate in transparent orthographies and two opaque orthographies, English and Chinese. All subjects were born and raised in Malaysia, a multilingual country in which Malay is the official language and English is the second official language. In public education, English and Malay are taught in all preschools from the age of five. Minority mother tongues such as Chinese and Tamil are taught at certain preschools, while Arabic is taught in religious classes. Therefore, all recruited subjects were at least bilingual in English and Malay. The subjects were right-handed and reported no neurological diseases. Demographic and language background details of the subjects are shown in Table 1.

**Table 1:** Participant demographic information and language background.

	Arabic Group: English-Malay-Arabic (*N* = 45)	Logographic Group:English-Malay-Mandarin (*N* = 45)	Latin Group: English-Malay (*N* = 45)
	Mean (SD)	Mean (SD)	Mean (SD)
Females (N)	29	35	40
Languages (N)	3.36 (0.53)	3.24 (0.53)	2.31 (0.60)
Age (years)	22.87 (3.55)	27.56 (5.46)	26.02 (5.15)
English AoA (years)	4.89 (2.18)	2.84 (2.82)	3.91 (2.43)
English Proficiency	0.76 (0.13)	0.78 (0.15)	0.82 (0.12)
SES	7.24 (2.52)	5.49 (2.54)	6.76 (2.33)

*N* = Number. AoA = Age of Acquisition. SES = Socioeconomic Status. English Proficiency ranged from 0 (No Proficiency) to 10 (High Proficiency). SES ranged from 1 (Low SES) to 10 (High SES).

### 4.2. Self-reported measures

#### Language Profile

The language backgrounds of the subjects were collected using a comprehensive questionnaire adapted from the Language and Social Background Questionnaire (LSBQ; Anderson et al., 2018). The adapted version can be found in Appendix A. It was administered on Gorilla Experiment Builder,^1^ an online platform for building behavioral experiments (Anwyl-Irvine et al., 2020). Important variables analyzed in conjunction with performance on the rhyme judgement task included age, English age of acquisition (AoA), English proficiency, number of languages, and socioeconomic status (SES). We used a 10-point Likert scale to rate proficiency for each language modality (Reading, Listening, Writing, and Speaking) and assigned equal weightages to each modality. Hence, overall proficiency scores for each language were calculated by summing individual self-rated proficiency levels before dividing by four. The highest level of education attained by both parents was used as a proxy for SES. To indicate the highest education level attained by each parent, subjects selected from a range of 1 (“No high school diploma”) to 5 (“Graduate or professional degree”). SES for each subject was calculated by summing ratings indicated for both parents. Hence, an overall rating of 1 represented low SES whereas 10 represented high SES. There were no significant differences in self-rated English proficiency between the three groups of participants [*F*(2, 134) = 2.82, *p* = 0.06]. There were significant differences in age [*F*(2, 134) = 11.20, *p* < 0.001], number of languages [*F*(2, 134) = 48.54, *p* < 0.001], SES [*F*(2, 134) = 6.10, *p* < 0.01], and English AoA [*F*(2, 134) = 7.59, *p* < 0.001] across the groups. The means and standard deviations of these variables for each group are shown in Table 1.

### 4.3. Rhyme Judgement task

#### Phonological Processing

**Figure 1:**
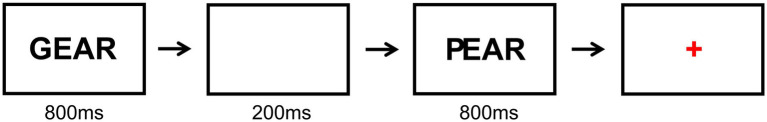
Illustration of a condition trial during the rhyme judgement task.

Participants performed a visual rhyme judgement task online in English *via* the data collection platform PsychoPy on Pavlovia.^2^ An example of a single trial is shown in Figure 1. The design of the rhyme judgement task was adapted from Kim et al. (2016). In this task, participants were shown word pairs one at a time on a white background and had to decide whether they rhymed as quickly and accurately as possible. They responded by pressing the left arrow key for “yes” and the right for “no.” This correspondence was counterbalanced. In each trial, the first word was shown for 800 ms followed by a 200 ms blank screen before the second word appeared for another 800 ms. A red fixation cross appeared after the offset of the second word, indicating that the subject should respond, after which the next trial was initiated. The fixation cross was presented for a maximum of 2,600 ms. There were 16 practice trials in which feedback was provided. A pilot session was conducted on a different group of subjects to assess task feasibility before adjustments were made to the version used in the study. In the previous version, the fixation cross that appeared after the offset of the second word remained on the screen for a fixed interval before the next trial appeared. This approach failed to detect response differences across the conditions, as the fixed response interval may have psychologically primed the individuals to not perform as quickly as they could. However, the approach used in the actual version of the task kept subjects alert, as their responses would trigger the onset of the next trial.

The rhyme judgement task consisted of four conditions: two rhyming and two non-rhyming. One of the rhyming conditions was congruent, consisting of word pairs that shared orthographic endings (O+P+); the other was incongruent, consisting of word pairs with different orthographic endings (O−P+). Of the two non-rhyming conditions, one was incongruent and consisted of similar orthographic endings (O+P−); the other was congruent with different orthographic endings (O−P−). The presentation order of the word pairs was counterbalanced across subjects. Examples of stimuli used in each condition are shown in Table 2. All stimuli had only one syllable and did not consist of homophones. They were matched across conditions for written word frequency [*F*(3, 175) = 0.44, *p* = 0.72], length [*F*(3, 175) = 1.91, *p* = 0.13], and number of phonemes [*F*(3, 175) = 1.54, *p* = 0.21] based on the lexical properties compiled in the English Lexicon Project database.^3^ The list of words used and their lexical properties can be found in Tables 7 and 8 in Appendix B.

**Table 2:** Examples of stimuli in each condition.

O+P+	O−P+	O+P−	O−P−
LINE/PINE	TALE/JAIL	GEAR/PEAR	CARD/SUIT

“O” refers to orthographically, “P” refers to phonologically, and “+” and “−” indicate similarity and difference, respectively. For example, O+P− means orthographically similar and phonologically different.

### 4.4. Statistical analysis

Subjects with accuracy rates lower than 60% for two or more conditions were considered to not have understood the task sufficiently and were excluded from the analysis. Inaccurate trials were removed from the analysis of RTs, comprising approximately 17.18% of the total number of trials. Outliers were identified as RTs showing more or less than two standard deviations from each subject’s mean, constituting approximately 5.1% of the total number of accurate trials. Each subject’s average performance in each condition was calculated and compared across groups and conditions. Univariate analyses (ANOVA) were performed on accuracy rates and RTs while controlling for sex, age, English proficiency, English AoA, number of languages, and SES by adding them as covariates. The means and standard deviations of these factors for each group are shown in Table 1. As these factors could potentially explain the variance in the dependent variables but were not of direct interest, they were added as covariates to reduce bias for a more precise model. We used an alpha level of 0.05 for all statistical tests. All post-hoc tests used Fisher’s least significance difference (LSD) technique.

## 5. Results

### 5.1. Rhyme judgement task performance: Accuracy

Among the covariates included in the by-participant analyses of accuracy, SES [*F_1_*(1, 522) = 23.68, *p* < 0.001, η^2^ = 0.04], AoA [*F_1_*(1, 522) = 4.66, *p* = 0.03, η^2^ = 0.009], and proficiency [*F_1_*(1, 522) = 8.77, *p* < 0.05, η^2^ = 0.02] were significantly related to accuracy, while age [*F_1_*(1, 522) = 1.79, *p* = 0.18, η^2^ = 0.003], number of languages [*F_1_*(1, 522) = 0.88, *p* = 0.35, η^2^ = 0.002], and sex [*F_1_*(1, 522) = 2.51, *p* = 0.11, η^2^ = 0.005] were not. After controlling for the covariates, there was no main effect of group [*F_1_*(2, 522) = 0.34, *p* = 0.71, η^2^ = 0.001; *F*_2_(2, 252) = 0.21, *p* = 0.81, η^2^ = 0.002] and no significant three-way interaction between group, phonological similarity, and orthographic similarity [*F_1_*(2, 522) = 0.58, *p* = 0.56, η^2^ = 0.002; *F_2_*(2, 252) = 0.94, *p* = 0.40, η^2^ = 0.007]. However, there was a main effect of phonological similarity on accuracy [*F_1_*(1, 522) = 91.07, *p* < 0.001, η^2^ = 0.15; *F_2_*(1, 252) = 154.80, *p* < 0.001, η^2^ = 0.38]. By-participant post-hoc tests showed that accuracy rates were higher when word pairs rhymed (*M* = 89.61, *SD* = 15.91) than when they did not (*M* = 76.03, *SD* = 27.05). Similarly, in the by-item analysis, responses were more accurate for rhyming word pairs (*M* = 89.14, *SD* = 8.51) than for non-rhyming word pairs (*M* = 74.83, *SD* = 23.01). There was also a main effect of orthographic similarity [*F_1_*(1, 522) = 95.64, *p* < 0.001, η^2^ = 0.16; *F_2_*(1, 252) = 162.67, *p* < 0.001, η^2^ = 0.39]. In the by-subject post-hoc tests, we found that accuracy was higher when word pairs did not share orthographic endings (*M* = 89.78, *SD* = 15.41) than when they did (*M* = 75.86, *SD* = 27.26). Similarly, in the post-hoc tests of the by-item analysis, accuracy was higher for orthographically different word pairs (*M* = 89.32, *SD* = 8.40) than for orthographically similar pairs (*M* = 74.65, *SD* = 22.93).

**Figure 2:**
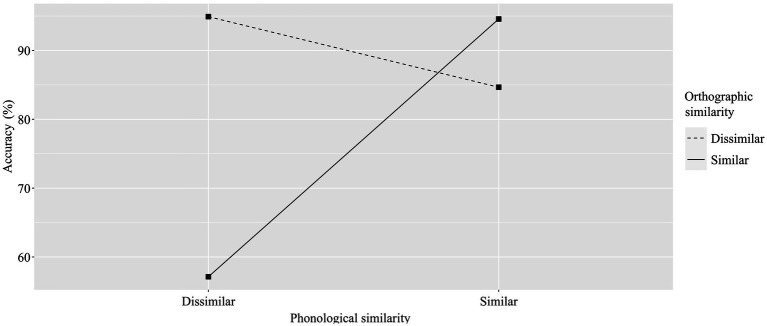
Effects of phonological and orthographic similarity on accuracy (%).

Additionally, there was a significant interaction between orthographic similarity and phonological similarity [*F_1_*(1, 522) = 280.11, *p* < 0.001, η^2^ = 0.35; *F_2_*(1, 252) = 477.80, *p* < 0.001, η^2^ = 0.66]. In the post-hoc tests of the by-subject analysis, we found that when word pairs rhymed, accuracy rates were significantly different between orthographically similar and dissimilar pairs (*p* < 0.001, 95% CI[5.96, 13.87]). Specifically, accuracy rates were higher for rhyming word pairs with similar orthographic endings (*M* = 94.58, *SD* = 12.67) than for rhyming pairs with different orthographic endings (*M* = 84.65, *SD* = 17.27). When word pairs did not rhyme, significant differences were observed between orthographically similar and dissimilar pairs (*p* < 0.001, 95% CI[33.82, 41.74]). Accuracy rates were higher for non-rhyming word pairs with different orthographic endings (*M* = 94.92, *SD* = 11.20) than for non-rhyming pairs with the same orthographic endings (*M* = 57.14, *SD* = 24.99). Similarly, in the by-item analysis, we found that when word pairs rhymed, accuracy rates differed significantly between orthographically similar and dissimilar pairs (*p* < 0.001, 95% CI[7.27, 13.67]). Accuracy rates were higher for rhyming word pairs with similar orthographic endings (*M* = 94.37, *SD* = 4.08) than for rhyming pairs with different orthographic endings (*M* = 83.90, *SD* = 8.58). When word pairs did not rhyme, significant differences were observed between orthographically similar and dissimilar pairs (*p* < 0.001, 95% CI[36.59, 43.00]). Accuracy rates were higher for non-rhyming word pairs with different orthographic endings (*M* = 94.73, *SD* = 3.01) than for non-rhyming pairs with the same orthographic endings (*M* = 54.93, *SD* = 15.93). See Figure 2 for a visualization of the results, Table 3 for by-participant means and standard deviations and Table 4 for by-item statistics. There were no separate interaction effects between group and phonological similarity [*F_1_*(2, 522) = 1.32, *p* = 0.27, η^2^ = 0.005; *F*_2_(2, 252) = 2.40, *p* = 0.09, η^2^ = 0.019] or between group and orthographic similarity [*F_1_*(2, 522) = 1.20, *p* = 0.30, η^2^ = 0.005; *F*_2_(2, 252) = 2.06, *p* = 0.13, η^2^ = 0.016].

**Table 3:** By-participant accuracy (%) means and standard deviations.

	O+		O−	
	P+	P−	P+	P−
Arabic group	95.15	53.64	85.35	95.86
	−11.6	−27.13	−15.89	−6.91
Latin group	94.95	57.07	86.67	94.65
	−13.26	−25.15	−12.98	−12.53
Logographic group	93.64	60.71	81.92	94.24
	−13.3	−22.54	−21.79	−13.27

The numbers in parentheses are the standard deviations. The N in all cases is 45. “O” refers to orthographically, “P” refers to phonologically, “+” refers to similar, and “−” refers to different.

**Table 4:** By-item accuracy (%) means and standard deviations.

	O+		O−	
	P+	P−	P+	P−
Arabic group	95	51.48	84.53	95.71
	−4.57	−15.46	−9.18	−3.03
Latin group	94.75	54.59	86.25	94.43
	−2.67	−16.38	−8.16	−2.82
Logographic group	93.35	58.74	80.92	94.05
	−4.68	−15.82	−7.86	−3.06

The numbers in parentheses are the standard deviations. “O” refers to orthographically, “P” refers to phonologically, “+” refers to similar, and “−” refers to different.

### 5.2. Rhyme judgement task performance: Reaction time

Among the covariates included in the by-participant analyses of RTs, age [*F_1_*(1, 521) = 6.14, *p* = 0.01, η^2^ = 0.012] and AoA [*F_1_*(1, 521) = 4.06, *p* = 0.04, η^2^ = 0.008] were significantly related to RTs, while number of languages [*F_1_*(1, 521) = 0.72, *p* = 0.40, η^2^ = 0.001], SES [*F_1_*(1, 521) = 3.00, *p* = 0.08, η^2^ = 0.006], proficiency [*F_1_*(1, 521) = 0.08, *p* = 0.77, η^2^ = 0.00], and sex [*F_1_*(1, 521) = 2.91, *p* = 0.09, η^2^ = 0.006] were not. After controlling for the effects of the covariates, there was a main effect of group [*F_1_*(2, 521) = 4.26, *p* = 0.02, η^2^ = 0.02; *F_2_*(2, 252) = 8.55, *p* < 0.001, η^2^ = 0.064], where the Arabic group (*M* = 371.76, *SD* = 142.84) had significantly slower RTs than both the Latin group (*p* = 0.01, 95% CI[11.96, 82.42], *M* = 344.47, *SD* = 116.03) and the logographic group (*p* = 0.02, 95% CI[6.78, 68.62], *M* = 348.33, *SD* = 126.84). Similarly, the by-item analysis showed that the Arabic group (*M* = 363.67, *SD* = 46.64) had slower RTs than both the Latin group (*p* < 0.01, 95% CI[12.58, 35.97], *M* = 339.40, *SD* = 43.15) and the logographic group (*p* = 0.01, 95% CI[3.60, 26.99], *M* = 348.37, *SD* = 61.65). There was a main effect of phonological similarity [*F_1_*(1, 521) = 11.83, *p <* 0.001, η^2^ = 0.02; *F_2_*(1, 252) = 49.15, *p* < 0.001, η^2^ = 0.16], where subjects were generally faster when the conditions rhymed (*M* = 336.83, *SD =* 106.04) than when they did not (*M* = 372.88, *SD* = 147.10). Similarly, in the by-item analysis, we found that the RTs for rhyming word pairs were faster (*M* = 333.49, *SD* = 39.38) than for non-rhyming pairs (*M* = 367.47, *SD* = 57.23). There was also a main effect of orthographic similarity [*F_1_*(1, 521) = 5.53, *p* = 0.02, η^2^ = 0.01; *F_2_* (1, 252) = 16.04, *p* < 0.001, η^2^ = 0.06], such that different orthographic endings led to faster RTs (*M* = 342.59, *SD* = 106.27) than similar orthographic endings (*M* = 367.10, *SD* = 148.13). Consistent with the findings from the by-participant analysis, the by-item analysis showed that orthographically different word pairs (*M* = 340.77, *SD* = 34.41) were responded to faster than orthographically similar word pairs (*M* = 360.19, *SD* = 63.52).

Additionally, the interaction effect between phonological and orthographic similarity was highly significant [*F_1_*(1, 521) = 30.36, *p* < 0.001, η^2^ = 0.06; *F_2_*(1, 252) = 115.24, *p* < 0.001, η^2^ = 0.31]. In the by-participant post-hoc tests, we found that when word pairs rhymed, orthographic similarity exerted a significant effect on RTs (*p* = 0.03, 95% CI[4.05, 62.81]). Specifically, seeing similar orthographic endings in rhyming word pairs led to faster responses (*M* = 320.03, *SD* = 94.49) than seeing different orthographic endings in rhyming pairs (*M* = 353.63, *SD* = 114.38). When word pairs did not rhyme, RTs between orthographically similar and dissimilar pairs were also significantly different (*p* < 0.001, 95% CI[53.77, 112.64]). RTs were much faster for non-rhyming pairs with different orthographic endings (*M* = 331.55, *SD* = 96.66) than for non-rhyming pairs with similar orthographic endings (*M* = 414.52, *SD* = 175.25). Similarly, in the by-item post-hoc tests, RTs for orthographically similar and dissimilar rhyming word pairs were significantly different (*p* < 0.001, 95% CI[19.13, 46.13]). RTs were faster for rhyming word pairs that shared orthographic endings (*M* = 317.17, *SD* = 36.24) than for rhyming pairs that did not (*M* = 349.80, *SD* = 35.66). Additionally, RTs differed significantly between non-rhyming word pairs with similar orthographic endings and non-rhyming pairs with different orthographic endings (*p* < 0.001, 95% CI[57.96, 84.96]). RTs were faster for non-rhyming word pairs with different orthographic endings (*M* = 331.74, *SD* = 30.81) than for non-rhyming pairs with similar orthographic endings (*M* = 403.20, *SD* = 55.32). See Table 5 for by-participant means and standard deviations.

**Table 5:** By-participant reaction time (ms) means and standard deviations.

	O+		O−	
	P+	P−	P+	P−
Arabic group	333.43	440.29	364.43	350.42
	−102.14	−191.92		135.15	−105.45
Latin group	314.01	399.46	338.07	326.33
	−80.18	−167.03	−95.06	−83.56
Logographic group	312.65	404.37	358.37	317.92
	−100.19	−167.13	−110.23	−98.84

The numbers in parentheses are the standard deviations. The N in all cases is 45. “O” refers to orthographically, “P” refers to phonologically, “+” refers to similar, and “−” refers to different.

**Figure 3:**
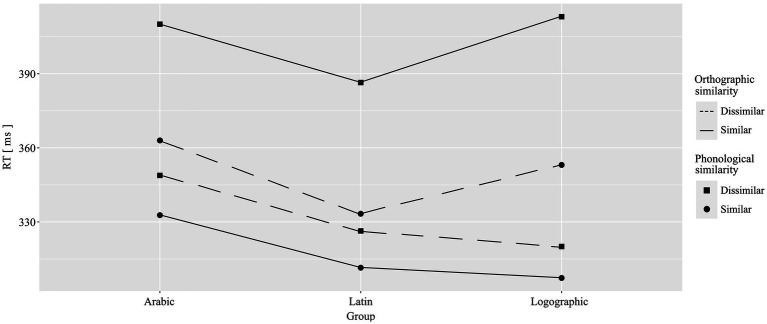
Effects of group, phonological similarity, and orthographic similarity on reaction time (ms).

Finally, there was a by-item three-way interaction between group, phonological similarity, and orthographic similarity [*F*_2_(2, 252) = 3.29, *p* = 0.04, η^2^ = 0.025; no effect by-participant, *F_1_*(2, 521) = 0.23, *p* = 0.79, η^2^ = 0.001]. The interaction patterns were such that when word pairs were orthographically and phonologically similar, the logographic group (*M* = 307.31, *SD* = 41.01) was significantly faster than the Arabic group (*M* = 332.74, *SD* = 34.13). Similarly, when word pairs were phonologically and orthographically different, the logographic group (*M* = 320.04, *SD* = 27.47) had shorter RTs than the Arabic group (*M* = 348.84, *SD* = 29.12). In the incongruent trials, when word pairs rhymed but were orthographically different, the Latin group (*M =* 333.32, *SD* = 29.42) was significantly faster than the Arabic group (*M* = 363.02, *SD* = 36.49). Additionally, when word pairs were orthographically similar but did not rhyme, the Latin group (*M* = 386.44, *SD* = 42.59) was faster than the logographic group (*M* = 413.10, *SD* = 70.93). See Table 6 for by-item statistics and Figure 3 for a visualization of the results. There were no separate interactions between group and phonological similarity [*F_1_*(2, 521) = 0.32, *p* = 0.73, η^2^ = 0.001; *F_2_*(2, 252) = 0.08, *p* = 0.92, η^2^ = 0.001] or between group and orthographic similarity [*F_1_*(2, 521) = 0.06, *p* = 0.94, η^2^ = 0.000; *F_2_*(2, 252) = 0.24, *p* = 0.79, η^2^ = 0.002].

**Table 6:** By-item reaction time (ms) means and standard deviations.

	O+		O−	
	P+	P−	P+	P−
Arabic group	332.74	410.08	363.02	348.84
	−34.13	−46.68	−36.49	−29.12
Latin group	311.47	386.44	333.32	326.35
	−28.79	−42.59	−29.42	−29.23
Logographic group	307.31	413.1	353.06	320.04
	−41.01	−70.93	−35.49	−27.47

The numbers in parentheses are the standard deviations. “O” refers to orthographically, “P” refers to phonologically, “+” refers to similar, and “−” refers to different.

## 6. Discussion

This study explored whether literacy in more opaque orthographies in a group of multilinguals influenced phonological awareness. This was done by assessing multilinguals who differed in their proportionate experience with opaque orthographies on a rhyme judgement task. The task consisted of four experimental conditions differing in terms of phonological and orthographic similarity: two congruent conditions (O+P+ and O−P−) and two incongruent conditions that required the processing of opaque pairs (O+P− and O−P+).

Results showed a main effect of group, where both the Latin and logographic groups were significantly faster than the Arabic group, which proportionately had the least opaque literacy experience. Additionally, the interaction effects showed that the Arabic group performed poorer in the O−P+, O+P+, and O−P− conditions than in either one or both the Latin and logographic groups. While better RTs could suggest better phonological representations (Andersson, 2002), the results do not clearly support the hypothesis that experience and practice with an additional opaque orthography apart from English enabled better performance on the rhyme judgement task. A clear indication of an advantage would manifest as significantly better performance in the group literate in opaque orthographies other than English (i.e., the logographic group). However, the results only partially support this by showing that the two groups with proportionately more opaque literacy experience (logographic and Latin groups) performed better than subjects whose language repertoire consisted of more transparent orthographies than opaque orthographies (Arabic group). The logographic group mainly consisted of individuals literate in Malay, English, and Chinese; thus, two-thirds of their languages have opaque orthographies. The Latin group consisted mostly of individuals literate in English and Malay; hence, half of their languages are opaque. In contrast, the Arabic group consisted of individuals whose only opaque orthography was in English. These results are in line with previous suggestions that orthographic knowledge could provide supplementary support in managing PA problems (Castles et al., 2003). Subjects in the opaque logographic group may have performed well because of their added experience with opaque orthographies as well as their exposure to orthographies across the transparency spectrum (in order of increasing orthographic opacity: Malay, English, and Chinese). Exposure to more speech sounds may have contributed to enhancing PA. Additionally, with Chinese being more opaque than English, the logographic group’s better performance over the Arabic group could suggest a cross-language transfer of PA from their more opaque orthography to a less opaque one. This would be in line with past bilingual studies and concepts of cross-language transfer, which predicts that when the shared linguistic feature is more apparent and complex in one of the reader’s two languages, it can simplify that of the other language. Additionally, the results support previous work, which showed that PA training in more opaque orthographies like Cantonese improved PA in less opaque orthographies like English (e.g., Chan, 2018). Furthermore, consistent with structural sensitivity theory (Kuo and Anderson, 2012), the opaque group’s exposure to proportionately more opaque orthographies throughout their lives would have given them additional opportunities to encounter more varied phonological segments, thus allowing them to rearrange linguistic input and assign linguistic structures more readily.

Past research has shown that linguistic skills from L1 are transferred to L2 and can affect the acquisition approach of the latter (Ben-Yehudah et al., 2019). With more practice in languages learned earlier, these languages are activated more effortlessly than newer languages. This could lead to individuals excessively applying linguistic rules, pronunciations, and transitional probabilities from their earlier language to later learned ones (Murphy, 2003), which may not always be appropriate. In this study, even though the AoA for English was added as a covariate in the analysis, English was the second language for many subjects in the Arabic group, while Malay was the first or home language. Unlike English, Malay has a highly transparent orthography, with regular GPCs. As such, the clear and consistent rules in Malay may have established an expectation of similarly regular GPC when learning a subsequent Latin-script language, such as English. This may have created a propensity for the subjects in this study to erroneously adopt it for English, which is their subsequent language, and could thus manifest as more mistakes in the task. In a similar vein, Frances et al. (2022) suggested that rules in the native language can negatively impact the process of mapping orthography to phonology in the subsequent language, especially if the native language is transparent while the subsequent language is opaque. Additionally, a quantitative study showed that at least 50% of the most common words in modern Malay are borrowed from Arabic (Zaidan et al., 2015). Subjects in the Arabic group may thus be more immersed in a more “transparent” literacy environment with explicit GPCs than the other groups. Furthermore, since English has more irregular mappings than Malay despite using the same Latin alphabet, subsequently acquiring English may be harder. Subjects would need to learn and “unlearn” the correspondences between the alphabetic letters and their pronunciations in Malay when acquiring English, and would find that the approach of matching graphemes to phonemes for Arabic and Malay does not work as effectively for English. Moreover, studies on neurobiological correlates in bilinguals show that acquiring a more opaque orthography taps into an accommodation strategy, which is more challenging and demands more neural resources (Shen and Del Tufo, 2022). On the other hand, the Latin group comprised simultaneous bilinguals who had been exposed to an equal mix of both transparent and opaque orthographies, and subjects in the logographic group had been exposed to more opaque orthographies. Such exposure for both groups may have served to better prepare them for inconsistencies in the English GPCs.

The results have also shown a facilitative effect of phonological similarity, where performance in the rhyming conditions was significantly faster and more accurate than in the non-rhyming conditions. The faster responses found in the rhyming conditions could be attributed to the first word of these pairs acting as a prime that facilitated the rhyme decision for the second word. This replicates results from earlier work with monolinguals and bilinguals of two languages differing in orthographic transparency (Botezatu et al., 2015; Sasisekaran and Lei, 2021). Such rhyming effects have also been found in studies conducted with event-related potentials, where rhyming targets are thought to be easier to process and require less cognitive resources, while non-rhyming targets tend to elicit more negative N400 amplitudes, which are related to exposure to unexpected events in a given context (Coch et al., 2008).

The main effect of orthographic similarity showed that RTs were better when word pairs did not share orthographic endings than when they did. Therefore, collectively, performance in trials that were orthographically similar regardless of rhyme (O+P+ and O+P−) was worse than in trials that were orthographically different (O−P+ and O−P−). However, the interaction effect found between orthographic similarity and phonological similarity indicated that the main effect of orthographic similarity was driven by superior performance in the congruent O−P− condition and poor performance in the incongruent O+P− condition. Additionally, results showed that orthographic similarity exerted a facilitative effect on rhyming word pairs but an inhibitory effect on non-rhyming pairs. This is consistent with previous studies (Damian and Bowers, 2010; Lipourli, 2014), which showed a similar “bias” in subjects where they were more likely to presume that words sharing orthographic endings would rhyme, while words with different orthographic endings would not (Weber-Fox et al., 2003; Tree et al., 2011). Furthermore, processing disruptions are most prominent when retrieving various phonological representations from the same orthographic symbols (Castro et al., 2003; Weber-Fox et al., 2003). Therefore, when subjects encountered word pairs that shared orthographic endings but were not sounded the same way, they may have felt compelled to check for rhymes once more, which could have slowed reaction times.

Notably, the interaction effect between phonological and orthographic similarity showed that subjects performed better in congruent conditions (O+P+ and O−P) and encountered greater difficulty in incongruent conditions (O+P− and O−P+). These incongruent conditions can occur due to the opacity of English orthography in both the feedforward and feedback directions, and this characteristic allows the language to be exploited in visual rhyme judgement tasks. The poorer performance in incongruent conditions underscores the influence of word opacity on reading performance (Ziegler et al., 1997), and is highly consistent with past studies conducted with both children and adults (Johnston and McDermott, 1986; Welcome and Alton, 2015). Unlike incongruent conditions, congruent conditions were not designed to activate other possible pronunciations, as the second word of each pair in these two congruent conditions makes it irrelevant to think of alternative pronunciations. This allows for shorter RTs, as subjects do not have to maneuver through GPC conflicts. For example, although-and is pronounced two ways depending on the word, its appearance in *hand* followed by *band* would not prompt subjects to think of its other pronunciation (e.g., *wand*). Additionally, *zone* activates the phonemes/o/and/n/, which are phonologically associated with many other words, like *tone*. Hence, in rhyming conditions with orthographically similar endings, the sound and form of the first word create a phonological expectation and thus facilitate recognition of the second word if it contains the same phonemes. Furthermore, with phonological codes activated in the first word, congruence in the orthography of the second word acts as confirmatory input.

In contrast, in incongruent conditions, the second word required subjects to either process the alternative sounds encoded by the same orthographic endings (O+P−) or identify the same sounds represented by different orthographic endings (O−P+). Such processes are more effortful and require additional time. For example, in the O−P+ condition, despite the activation of phonological codes in processing the first word, the orthographic expectations of the second word were not met. The conflicting orthographic input of the second word required subjects to decode a new set of graphemes and revisit the phonological representation of the first word to match it, extending processing time. Between incongruent conditions, RTs were better for rhyming word pairs that were orthographically different (O−P+) than for non-rhyming word pairs that looked similar (O+P−). The O−P+ condition may be easier than the O+P− condition because seeing the first word (e.g., *white*) activates phonological codes shared with other words (e.g., *light*). This could create an expectation for a phonological match, and in turn facilitate rhyme judgement in the O−P+ condition (see Hillinger, 1980).

However, this explanation would not apply to the O+P− condition, as the first word would have triggered phonological codes that were violated by the second word. To make a rhyme judgement in such a condition, subjects would have to tap into their lexical knowledge and recode the same symbols in a different set of phonological codes. Orthographic similarities would thus contribute to phonological interference, leading to increased error rates and response latencies. The challenge experienced in the O+P− condition is consistent with past behavioral and neuroimaging studies (Kramer and Donchin, 1987; Levinthal and Hornung, 1992; Bitan et al., 2009). Furthermore, Bitan et al. (2009) suggested that reading an opaque word entailed repeated matching of orthography to phonology, as well as better phonological segmentation skills. Particularly for non-rhyming word pairs with matching orthographies, the word presented first acts as a prime with different phonological codes. Therefore, the likelihood of repeated matching is said to increase, consequently eliciting additional effort on the reader’s part. The difficulty in processing non-rhyming word pairs with similar orthography is also supported by neuroimaging findings, which revealed increased activation in two brain regions involved in conflict resolution and phonological access.

### 6.1. Limitations

One limitation is the use of self-reported language proficiency, which may not be an accurate reflection of the subjects’ actual language abilities in the various domains. Although these self-ratings were simple to collect, they may be vulnerable to subjectivity, such as positivity bias. This is especially tricky in a multilingual population, since it has previously been found that self-reports of L1 proficiency are more objective than of L2 proficiency (see Marian et al., 2007). Future studies should consider administering objective measures of language proficiency. Second, while the three groups differed in the transparency of their orthographies, they are also literate in different types of scripts, which may have also contributed to the findings. Comparing multiliterate groups that share the same script but differ in orthographic transparency (i.e., Italian-Spanish-English multiliterate individuals compared with Italian-Spanish-Dutch multiliterate individuals) may be a potential direction for future work. Another limitation relates to individual variability in the subject groups. The subjects in this study vary in other factors, such as AoA and phonological repertoires, and they may be exposed to an undefined range of linguistic influences across different platforms. Although mitigation steps were taken by including potential variables as covariates, these factors may have also contributed to the findings. That said, investigating a highly multilingual and multiliterate population in a linguistically rich setting poses a unique set of challenges that makes it difficult, if not impossible to account for every potential factor.

### 6.2. Conclusion

To our best knowledge, this is the first study uniquely exploring rhyme judgement in an understudied multiliterate population. We examined a population consisting of individuals literate in a variety of orthographies that differ in orthographic transparency. Apart from highlighting findings consistent with other visual rhyme judgement studies, the results also suggest that rhyming ability could be an ongoing process where literacy experience acquired over time may modify performance on PA tasks. Although our findings are preliminary and we did not find conclusive evidence for the influence of opaque literacy experience on phonological awareness, this study has provided a novel research direction for further exploration of the interactions between orthographic transparency and phonological processing within multiliterate and multilingual populations.

## Data availability statement

The raw data supporting the conclusions of this article are included in the Supplementary material, further inquiries can be directed to the corresponding author.

## Ethics statement

The studies involving human participants were reviewed and approved by the Ethics Committee for Research Involving Human Subjects at Universiti Putra Malaysia (JKEUPM2020-272). The patients/participants provided their written informed consent to participate in this study.

## Author contributions

NY and JY: conceptualization, design, and writing – review and editing. JY: methodology – data acquisition, preparation, and analysis, formal analysis, investigation, and writing – original draft preparation. NY: funding acquisition. NY, RM, and MS: supervision. All authors contributed to the article and approved the submitted version.

## Funding

The research leading to these results has received funding from the European Union’s Horizon2020 research and innovation program under Marie Skłodowska-Curie grant agreement No. 765556.

## Conflict of interest

The authors declare that the research was conducted in the absence of any commercial or financial relationships that could be construed as a potential conflict of interest.

## Publisher’s note

All claims expressed in this article are solely those of the authors and do not necessarily represent those of their affiliated organizations, or those of the publisher, the editors and the reviewers. Any product that may be evaluated in this article, or claim that may be made by its manufacturer, is not guaranteed or endorsed by the publisher.

## Footnotes

^1^ www.gorilla.sc

^2^ https://pavlovia.org/

^3^ https://elexicon.wustl.edu/
