# Supplementary material for: Effects of orthographic transparency on rhyme judgement
Source: Front Psychol. 2023 Mar 6;14:1038630. doi: 10.3389/fpsyg.2023.1038630 (PMC10026565; doi:10.3389/fpsyg.2023.1038630)
Supplement: Supplementary file 2 [file Data_Sheet_2.docx]

# Appendix A

**Language Background Questionnaire**

– Adapted from the LSBQ (Anderson, Mak, Keyvani Chahi, & Bialystok, 2018)

Today’s Date: _________________ (dd/mm/yy)

**1.** Sex: Male **o** Female **o**

1. Occupation/Student Status (i.e. FT/PT, current year of study): ____________________
2. Handedness: Left **o** Right **o**
3. Date of Birth: ___________________ (dd/mm/yy)
4. Do you play first-person shooting (FPS/action video games? **Yes o No o**
    If **yes,** on average how many hours do you play per week?
5. Do you have hearing problems? **Yes o No o**

If **yes,** do you wear a hearing aid? ____________

1. Do you have vision problems? **Yes o No o**
    If **yes,** do you wear glasses or contact lenses? ______________
2. Are you colour-blind? **Yes o No o**
    If **yes,** what type? _____________________________________
3. Have you ever had a head injury? **Yes o No o**
    If **yes,**  please explain: _______________________________________
4. Do you have any known neurological impairments? **Yes o No o**(e.g. epilepsy etc.)
5. Are you currently taking any psychoactive medications? **Yes o No o**

**12**. Please indicate the highest level of education and occupation for each parent:

| **Mother** | **Father** |
| --- | --- |
| 1. _____ No high school diploma | 1. _____ No high school diploma |
| 1. _____ High school diploma | 2. _____ High school diploma |
| 1. _____ Some post-secondary education | 3. _____ Some post-secondary education |
| 1. _____ Post-secondary degree/Diploma | 4. _____ Post-secondary degree/Diploma |
| 1. _____ Graduate or professional degree | 5. _____ Graduate or professional degree |
| Occupation: | Occupation: |
| First Language: | First Language: |
| Second Language: | Second Language: |
| Other Language | Other Language: |

**13.** Were you born in Malaysia? **Yes o No o**

If **not**, where were you born? ___________________________

When did you move to Malaysia? ___________________________ (Year)

**14.** Have you ever lived in a place where Malay/English is not the dominant
 communicating language? **Yes o No o**

**From (Year) To (Year)**

| If **yes**, where and for how long? | 1. |  |  |
| --- | --- | --- | --- |
|  | 2. |  |  |
|  | 3. |  |  |

**Language Background**

**15.** List all the languages and dialects you can speak and understand including English, ***in order of fluency:***

| Language | Where did you learn it? | At what age did you learn it? (if learned from birth, write age “0”) | Were there any periods in your life when you did not use this language? Indicate duration in months/years |
| --- | --- | --- | --- |
|  | **o Home o School**  **o Community**  **o Other: _________** |  |  |
|  | **o Home o School**  **o Community**  **o Other: _________** |  |  |
|  | **o Home o School**  **o Community**  **o Other: _________** |  |  |
|  | **o Home o School**  **o Community**  **o Other: _________** |  |  |
|  | **o Home o School**  **o Community**  **o Other: _________** |  |  |

**16.1** Relative to a highly proficient speaker’s performance, **rate your proficiency level** on a scale of 0-10 for the following activities conducted in English and your other language(s):

| **English** | |  | |
| --- | --- | --- | --- |
| No Proficiency | |  | High Proficiency |
|  | 0 | 5 | 10 |
| Speaking | 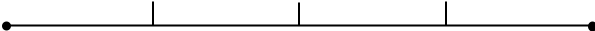  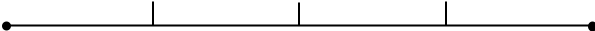  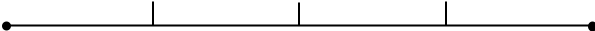  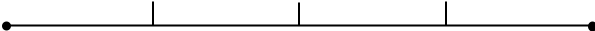 | | |
| Understanding |  |  |  |
| Reading |  |  |  |
| Writing |  |  |  |

**16.2** Of the time you spend engaged in each of the following activities, how much of that time is carried out in **English**?

|  | None | Little | Some | Most | All |
| --- | --- | --- | --- | --- | --- |
| Speaking | o | o | o | o | o |
| Listening | o | o | o | o | o |
| Reading | o | o | o | o | o |
| Writing | o | o | o | o | o |

| **17.1** **Other Language: ___________________** | |  | |
| --- | --- | --- | --- |
| No Proficiency | |  | High Proficiency |
|  | 0 | 5 | 10 |
| Speaking | 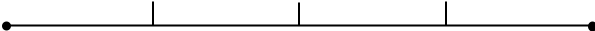  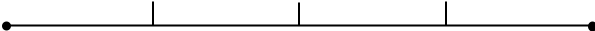  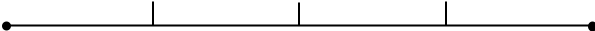  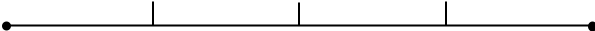 | | |
| Understanding |  |  |  |
| Reading |  |  |  |
| Writing |  |  |  |

**17.2** Of the time you spend engaged in each of the following activities, how much of that time is carried out in this language?

|  | None | Little | Some | Most | All |
| --- | --- | --- | --- | --- | --- |
| Speaking | o | o | o | o | o |
| Listening | o | o | o | o | o |
| Reading | o | o | o | o | o |
| Writing | o | o | o | o | o |

| **18.1** **Other Language:**  **___________________** | |  | |
| --- | --- | --- | --- |
| No Proficiency | |  | High Proficiency |
|  | 0 | 5 | 10 |
| Speaking | 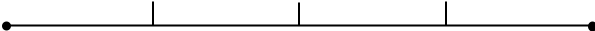  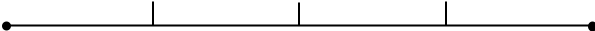  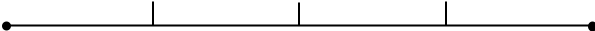  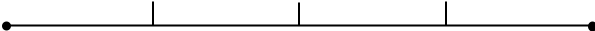 | | |
| Understanding |  |  |  |
| Reading |  |  |  |
| Writing |  |  |  |

**18.2** Of the time you spend engaged in each of the following activities, how much of that time is carried out in this language?

|  | None | Little | Some | Most | All |
| --- | --- | --- | --- | --- | --- |
| Speaking | o | o | o | o | o |
| Listening | o | o | o | o | o |
| Reading | o | o | o | o | o |
| Writing | o | o | o | o | o |

| **19.1** **Other Language: ______________________** | |  | |
| --- | --- | --- | --- |
| No Proficiency | |  | High Proficiency |
|  | 0 | 5 | 10 |
| Speaking | 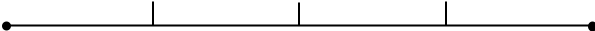  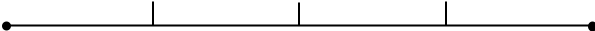  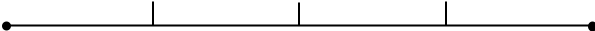  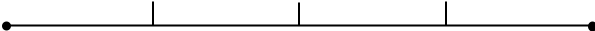 | | |
| Understanding |  |  |  |
| Reading |  |  |  |
| Writing |  |  |  |

**19.2** Of the time you spend engaged in each of the following activities, how much of that time is carried out in this language?

|  | None | Little | Some | Most | All |
| --- | --- | --- | --- | --- | --- |
| Speaking | o | o | o | o | o |
| Listening | o | o | o | o | o |
| Reading | o | o | o | o | o |
| Writing | o | o | o | o | o |

**Community Language Use Behaviour**

**20.** Please indicate the proportion of the language(s) you most frequently heard or
 used in the following life stages, both inside and outside home:

|  |  | English | Malay | Chinese | Other language: ________ | Other language: _______ | Total percentage |
| --- | --- | --- | --- | --- | --- | --- | --- |
| 20.1 | Infancy |  |  |  |  |  | 100% |
| 20.2 | Preschool age |  |  |  |  |  | 100% |
| 20.3 | Primary School age |  |  |  |  |  | 100% |
| 20.4 | Secondary School age |  |  |  |  |  | 100% |

**21.** Please indicate which language(s) you generally use when speaking to the following
 people:

|  |  | English | Malay | Chinese | Other language: _________ | Other language: ________ | Total  Percentage |
| --- | --- | --- | --- | --- | --- | --- | --- |
| 21.1 | Parents |  |  |  |  |  | 100% |
| 21.2 | Siblings |  |  |  |  |  | 100% |
| 21.3 | Grandparents |  |  |  |  |  | 100% |
| 21.4 | Other Relatives |  |  |  |  |  | 100% |
| 21.5 | Partner |  |  |  |  |  | 100% |
| 21.6 | Roommates |  |  |  |  |  | 100% |
| 21.7 | Neighbours |  |  |  |  |  | 100% |
| 21.8 | Friends |  |  |  |  |  | 100% |

**22.** Please indicate which language(s) you generally use in the following situations:

|  |  | English | Malay | Chinese | Others: _______ | Others:  ______ | Total  Percentage |
| --- | --- | --- | --- | --- | --- | --- | --- |
| 22.1 | Home |  |  |  |  |  | 100% |
| 22.2 | School |  |  |  |  |  | 100% |
| 22.3 | Work |  |  |  |  |  | 100% |
| 22.4 | Social activities (e.g. hanging out with friends, movies) |  |  |  |  |  | 100% |
| 22.5 | Religious activities |  |  |  |  |  | 100% |
| 22.6 | Extracurricular activities (e.g. hobbies, sports, volunteering, gaming) |  |  |  |  |  | 100% |
| 22.7 | Shopping/ Restaurants/Other commercial services |  |  |  |  |  | 100% |
| 22.8 | Healthcare services/ Government/ Public offices/ Banks |  |  |  |  |  | 100% |

**23.** Please indicate which language(s) you generally use for the following activities:

|  |  | English | Malay | Chinese | Others: ______ | Others: ______ | Total  Percentage |
| --- | --- | --- | --- | --- | --- | --- | --- |
| 23.1 | Reading |  |  |  |  |  | 100% |
| 23.2 | Emailing |  |  |  |  |  | 100% |
| 23.3 | Work |  |  |  |  |  | 100% |
| 23.4 | Social media (e.g. Facebook, Twitter etc) |  |  |  |  |  | 100% |
| 23.5 | Writing shopping lists, notes, etc |  |  |  |  |  | 100% |
| 23.6 | Watching TV/ listening to radio |  |  |  |  |  | 100% |
| 23.7 | Watching movies |  |  |  |  |  | 100% |
| 23.8 | Browsing on the internet |  |  |  |  |  | 100% |
| 23.9 | Praying |  |  |  |  |  | 100% |

**24.** Some people **switch between the languages they know within a single
 conversation** (i.e. while speaking in one language they may use sentences or words
 from the other language). This is known as “language-switching”. Please indicate
 how often you engage in language-switching:

|  |  | Never | Rarely | Sometimes | Frequently | Always |
| --- | --- | --- | --- | --- | --- | --- |
| 24.1 | With parents and family | o | o | o | o | o |
| 24.2 | With friends | o | o | o | o | o |
| 24.3 | On social media (e.g. Facebook, Twitter) | o | o | o | o | o |

# Appendix B

**Table 7. List of words for each condition in the rhyme judgement task**

| **Orthographically similar and**  **Phonologically similar**  **O+P+** | | **Orthographically different and phonologically similar**  **O-P+** | | **Orthographically similar and phonologically different**  **O+P-** | | **Orthographically different and phonologically different**  **O-P-** | |
| --- | --- | --- | --- | --- | --- | --- | --- |
| **Word 1** | **Word 2** | **Word 1** | **Word 2** | **Word 1** | **Word 2** | **Word 1** | **Word 2** |
| MATE | GATE | JAZZ | HAS | PINT | MINT | CLAP | LIST |
| FLOSS | CROSS | DUTCH | MUCH | SLUSH | BUSH | TAPE | SLOPE |
| BAND | HAND | MALE | SAIL | HEAD | BEAD | DRAIN | PRICE |
| FAIR | HAIR | TALE | JAIL | WORD | CORD | SOUP | WISH |
| DANCE | CHANCE | VERB | CURB | POST | LOST | CREST | GLASS |
| DENT | VENT | BREAK | SHAKE | TOUR | FOUR | CRIME | GRAIN |
| LUNCH | HUNCH | WAIST | PASTE | WAND | SAND | DIRT | FISH |
| DOOR | FLOOR | FAME | CLAIM | BEAR | FEAR | FACE | HOUSE |
| LUCK | DUCK | SEAM | DEEM | COST | MOST | NOTE | TRAY |
| FISH | WISH | GENES | BEANS | REAL | SEAL | BIRD | PATCH |
| TEAM | BEAM | FORCE | HORSE | BULL | DULL | BALM | SCENE |
| ROPE | HOPE | BAIT | DATE | FIEND | FRIEND | CARD | SUIT |
| FAME | NAME | GAIT | FATE | PATCH | WATCH | RACK | GUEST |
| FANG | GANG | MEAT | SHEET | BROW | SNOW | BEAM | SMOKE |
| DICE | MICE | SITE | MIGHT | HOWL | BOWL | LAWN | DREAM |
| FIRE | WIRE | FARE | PAIR | GEAR | PEAR | FILE | ROCK |
| RUST | DUST | DONE | FUN | FOUL | SOUL | LEAF | FORT |
| TART | PART | BEAN | GREEN | COMB | BOMB | NEWS | FOAM |
| BONE | TONE | CHORD | BOARD | GILD | MILD | DECK | DRAFT |
| SOOT | FOOT | SEAT | FEET | HOST | FROST | CRISP | RANGE |
| LINE | PINE | CRAZE | PHASE | PLANT | RANT | FIELD | CHART |
| WOOD | HOOD | DEAL | HEEL | DOUGH | COUGH | FERN | MAZE |

**Table 8. Frequency, length, and number of phonemes of each word in all conditions**

| **Condition** | **Word** | **Frequency** | **Length** | **Number of phonemes** |
| --- | --- | --- | --- | --- |
| **O+P+** | BAND | 10.803 | 4 | 4 |
|  | BEAM | 8.972 | 4 | 3 |
|  | BONE | 9.684 | 4 | 3 |
|  | CHANCE | 10.985 | 6 | 4 |
|  | CROSS | 10.301 | 5 | 4 |
|  | DANCE | 10.171 | 5 | 4 |
|  | DENT | 7.81 | 4 | 4 |
|  | DICE | 9.609 | 4 | 4 |
|  | DOOR | 10.893 | 4 | 3 |
|  | DUCK | 8.829 | 4 | 3 |
|  | DUST | 9.833 | 4 | 4 |
|  | FAIR | 10.7 | 4 | 3 |
|  | FAME | 8.714 | 4 | 3 |
|  | FANG | 6.821 | 4 | 3 |
|  | FIRE | 11.046 | 4 | 3 |
|  | FISH | 10.329 | 4 | 3 |
|  | FLOOR | 10.481 | 5 | 4 |
|  | FLOSS | 7.126 | 5 | 4 |
|  | FOOT | 10.095 | 4 | 3 |
|  | GANG | 8.965 | 4 | 3 |
|  | GATE | 9.719 | 4 | 3 |
|  | HAIR | 10.66 | 4 | 3 |
|  | HAND | 11.735 | 4 | 4 |
|  | HOOD | 8.931 | 4 | 3 |
|  | HOPE | 11.923 | 4 | 3 |
|  | HUNCH | 6.594 | 5 | 4 |
|  | LINE | 12.388 | 4 | 3 |
|  | LUCK | 11.013 | 4 | 3 |
|  | LUNCH | 9.314 | 5 | 4 |
|  | MATE | 8.552 | 4 | 3 |
|  | MICE | 8.338 | 4 | 3 |
|  | NAME | 12.604 | 4 | 3 |
|  | PART | 12.425 | 4 | 4 |
|  | PINE | 8.977 | 4 | 3 |
|  | ROPE | 8.757 | 4 | 3 |
|  | RUST | 8.34 | 4 | 4 |
|  | SOOT | 5.645 | 4 | 3 |
|  | TART | 6.354 | 4 | 4 |
|  | TEAM | 11.595 | 4 | 3 |
|  | TONE | 9.604 | 4 | 3 |
|  | VENT | 7.629 | 4 | 4 |
|  | WIRE | 9.718 | 4 | 3 |
|  | WISH | 11.216 | 4 | 3 |
|  | WOOD | 10.087 | 4 | 3 |
| **O-P+** | BAIT | 8.364 | 4 | 3 |
|  | BEAN | 8.182 | 4 | 3 |
|  | BEANS | 8.487 | 5 | 4 |
|  | BOARD | 11.285 | 5 | 4 |
|  | BREAK | 10.774 | 5 | 4 |
|  | CHORD | 8.352 | 5 | 4 |
|  | CLAIM | 10.944 | 5 | 4 |
|  | CRAZE | 6.541 | 5 | 4 |
|  | CURB | 7.622 | 4 | 3 |
|  | DATE | 11.822 | 4 | 3 |
|  | DEAL | 11.336 | 4 | 3 |
|  | DEEM | 7.006 | 4 | 3 |
|  | DONE | 12.067 | 4 | 3 |
|  | DUTCH | 9.032 | 5 | 3 |
|  | FAME | 8.714 | 4 | 3 |
|  | FARE | 8.577 | 4 | 3 |
|  | FATE | 9.047 | 4 | 3 |
|  | FEET | 10.6 | 4 | 3 |
|  | FORCE | 11.23 | 5 | 4 |
|  | FUN | 11.234 | 3 | 3 |
|  | GAIT | 6.485 | 4 | 3 |
|  | GENES | 8.639 | 5 | 4 |
|  | GREEN | 11.416 | 5 | 4 |
|  | HAS | 13.978 | 3 | 3 |
|  | HEEL | 8.384 | 4 | 3 |
|  | HORSE | 10.08 | 5 | 4 |
|  | JAIL | 9.176 | 4 | 3 |
|  | JAZZ | 9.529 | 4 | 3 |
|  | MALE | 10.978 | 4 | 3 |
|  | MEAT | 9.606 | 4 | 3 |
|  | MIGHT | 12.534 | 5 | 3 |
|  | MUCH | 13.09 | 4 | 3 |
|  | PAIR | 10.053 | 4 | 3 |
|  | PASTE | 8.803 | 5 | 4 |
|  | PHASE | 9.726 | 5 | 3 |
|  | SAIL | 8.175 | 4 | 3 |
|  | SEAM | 6.989 | 4 | 3 |
|  | SEAT | 9.752 | 4 | 3 |
|  | SHAKE | 8.574 | 5 | 3 |
|  | SHEET | 9.742 | 5 | 3 |
|  | SITE | 11.91 | 4 | 3 |
|  | TALE | 8.733 | 4 | 3 |
|  | VERB | 7.65 | 4 | 3 |
|  | WAIST | 8.571 | 5 | 4 |
| **O+P-** | BEAD | 7.509 | 4 | 3 |
|  | BEAR | 10.066 | 4 | 3 |
|  | BOMB | 9.641 | 4 | 3 |
|  | BOWL | 9.28 | 4 | 3 |
|  | BROW | 6.69 | 4 | 3 |
|  | BULL | 9.025 | 4 | 3 |
|  | BUSH | 9.296 | 4 | 3 |
|  | COMB | 7.386 | 4 | 3 |
|  | CORD | 8.441 | 4 | 4 |
|  | COST | 11.508 | 4 | 4 |
|  | COUGH | 7.703 | 5 | 3 |
|  | DOUGH | 8.129 | 5 | 2 |
|  | DULL | 8.443 | 4 | 3 |
|  | FEAR | 10.45 | 4 | 3 |
|  | FIEND | 6.987 | 5 | 4 |
|  | FOUL | 8.312 | 4 | 3 |
|  | FOUR | 11.353 | 4 | 3 |
|  | FRIEND | 11.303 | 6 | 5 |
|  | FROST | 8.708 | 5 | 5 |
|  | GEAR | 9.637 | 4 | 3 |
|  | GILD | 4.344 | 4 | 4 |
|  | HEAD | 11.6 | 4 | 3 |
|  | HOST | 10.474 | 4 | 4 |
|  | HOWL | 8.079 | 4 | 3 |
|  | LOST | 11.266 | 4 | 4 |
|  | MILD | 8.582 | 4 | 4 |
|  | MINT | 11.344 | 4 | 4 |
|  | MOST | 13.113 | 4 | 4 |
|  | PATCH | 10.136 | 5 | 3 |
|  | PEAR | 6.878 | 4 | 3 |
|  | PINT | 7.293 | 4 | 4 |
|  | PLANT | 10.136 | 5 | 5 |
|  | POST | 12.528 | 4 | 4 |
|  | RANT | 7.959 | 4 | 4 |
|  | REAL | 12.195 | 4 | 4 |
|  | SAND | 9.219 | 4 | 4 |
|  | SEAL | 8.767 | 4 | 3 |
|  | SLUSH | 5.861 | 5 | 4 |
|  | SNOW | 9.914 | 4 | 3 |
|  | SOUL | 10.384 | 4 | 3 |
|  | TOUR | 10.064 | 4 | 3 |
|  | WAND | 8.598 | 4 | 4 |
|  | WATCH | 10.95 | 5 | 3 |
|  | WORD | 11.836 | 4 | 3 |
| **O-P-** | BALM | 7.321 | 4 | 3 |
|  | BEAM | 8.972 | 4 | 3 |
|  | BIRD | 9.856 | 4 | 3 |
|  | CARD | 12.314 | 4 | 4 |
|  | CHART | 9.274 | 5 | 4 |
|  | CLAP | 6.837 | 4 | 4 |
|  | CREST | 7.162 | 5 | 5 |
|  | CRIME | 10.419 | 5 | 4 |
|  | CRISP | 7.619 | 5 | 5 |
|  | DECK | 10.267 | 4 | 3 |
|  | DIRT | 8.941 | 4 | 3 |
|  | DRAFT | 9.557 | 5 | 5 |
|  | DRAIN | 9.606 | 5 | 4 |
|  | DREAM | 10.387 | 5 | 4 |
|  | FACE | 11.261 | 4 | 3 |
|  | FERN | 6.344 | 4 | 3 |
|  | FIELD | 11.289 | 5 | 4 |
|  | FILE | 12.65 | 4 | 3 |
|  | FISH | 10.329 | 4 | 3 |
|  | FOAM | 8.205 | 4 | 3 |
|  | FORT | 9.038 | 4 | 4 |
|  | GLASS | 9.941 | 5 | 4 |
|  | GRAIN | 8.791 | 5 | 4 |
|  | GUEST | 9.604 | 5 | 4 |
|  | HOUSE | 11.554 | 5 | 3 |
|  | LAWN | 7.887 | 4 | 3 |
|  | LEAF | 8.861 | 4 | 3 |
|  | LIST | 12.667 | 4 | 4 |
|  | MAZE | 8.775 | 4 | 3 |
|  | NEWS | 11.912 | 4 | 3 |
|  | NOTE | 11.788 | 4 | 3 |
|  | PATCH | 10.136 | 5 | 3 |
|  | PRICE | 11.943 | 5 | 4 |
|  | RACK | 9.363 | 4 | 3 |
|  | RANGE | 10.971 | 5 | 4 |
|  | ROCK | 10.698 | 4 | 3 |
|  | SCENE | 10.371 | 5 | 3 |
|  | SLOPE | 7.897 | 5 | 4 |
|  | SMOKE | 9.72 | 5 | 4 |
|  | SOUP | 8.707 | 4 | 3 |
|  | SUIT | 9.813 | 4 | 3 |
|  | TAPE | 11.021 | 4 | 3 |
|  | TRAY | 8.126 | 4 | 3 |
|  | WISH | 11.216 | 4 | 3 |

*Note.* “O” refers to orthographically, “P” refers to phonologically, “+” refers to similar, and “-” refers to different.
